# A Novel Delivery System for the Combined Use of Natural Ingredients: The Preparation of Berberine Hydrochloride–Matrine Liposomes and Preliminary Exploration of Their Anti-Tumor Activity

**DOI:** 10.3390/molecules29215210

**Published:** 2024-11-04

**Authors:** Min Xu, Zhangkai Ye, JunJing Liu, Shunpeng Zhu, Yuchen Chen, Jia Cai, Yangxi Chen, Long Wang, Liang Zhang, Qiang Ye

**Affiliations:** 1 State Key Laboratory of Southwestern Chinese Medicine Resources, Chengdu University of Traditional Chinese Medicine, Chengdu 611137, China; 18381083126@163.com (M.X.); 19182068627@163.com (J.L.); zhushunpeng@stu.cdutcm.edu.cn (S.Z.); chenyuchen@stu.cdutcm.edu.cn (Y.C.); caijia@stu.cdutcm.edu.cn (J.C.); a18081033128@163.com (Y.C.); 18523939143@163.com (L.W.); 2 College of Pharmacy, School of Modern Chinese Medicine Industry, Chengdu University of Traditional Chinese Medicine, Chengdu 611137, China; 3 Xinjiang Normal University Business School, Xinjiang Normal University, Urumqi 830017, China; yeah198101@163.com; 4 Chengdu Institute for Drug Control, Chengdu 610000, China

**Keywords:** natural compounds, berberine hydrochloride, matrine, drug combined use, liposomes, anti-tumor activity, enhancement of efficacy, slow sustained release

## Abstract

Berberine hydrochloride (BH) extracted from Coptis chinensis (CC) and Matrine (MT) separated from Sophora flavescens (SF) are alkaloids with potent anti-bacterial, anti-inflammatory, and anti-tumor effects. Motivated by the clinical practice of using CC and SF together, we aimed to demonstrate that the synergistic application of the natural compounds BH and MT could enhance therapeutic effects and minimize side effects. Two types of liposomes, liposomes containing only BH (BH-LP) and liposomes containing both BH and MT (BH-MT-LP), were successfully prepared via the reverse evaporation method. The liposome preparation process was optimized by single-factor screening and the Box–Behnken experimental design method. The results showed that the liposomes had particle sizes in the range of 222.7 to 235.4 nm, polydispersity indicated in the range of 11.8% to 23.3%, and zeta potentials in the range of −35.9 to −31.1 mv. BH-MT-LP showed superior anti-tumor activity against MDA-MB-231, HepG-2, and HGC-27 cells in vitro. The incorporation of MT effectively promoted the anti-tumor effect of BH, while the controlled release from liposomes further enhanced the therapeutic efficacy of BH. Furthermore, based on the flow cytometry results, we speculated that BH-MT-LP might promote apoptosis by blocking the G1 phase of cells and inducing cell death. In conclusion, BH-MT-LP provides evidence for the combined use of natural compounds as a stable, safe, and practical drug delivery system for the treatment of potential cancers. Meanwhile, the successful preparation for BH-MT-LP also provides a new approach to the combined use of traditional Chinese medicine ingredients.

## 1. Introduction

**Figure 1:**
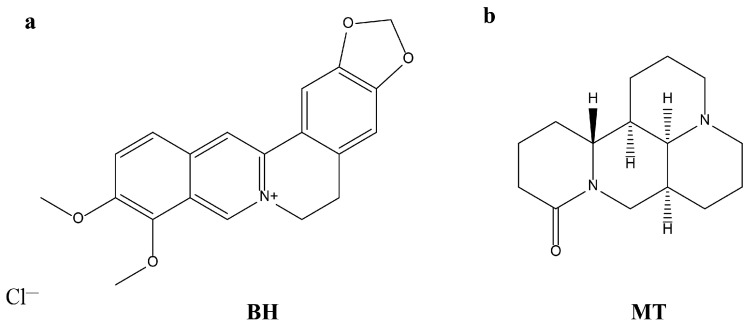
Chemical structures of Berberine hydrochloride and Matrine. BH (**a**), MT (**b**).

Traditional Chinese medicines (TCMs) contain numerous naturally active compounds, so Chinese people always use a variety of herbal extracts to treat a wide range of ailments, from simple infections to cardiovascular disease [1]. Natural compounds extracted from plants have pharmacological activity, and the combined use of drugs can enhance pharmacological activity, which has become a promising method for clarifying and developing TCMs [2]. Coptis chinensis (CC) is listed as a top-grade herb in the Shen-nong Materia Medica Classic, with the effects of clearing heat, drying dampness, purging fire, and detoxification. Sophora flavescens (SF) has the effects of clearing heat, drying dampness, killing insects, and diuresis [3,4,5]. In clinical practice, CC and SF are often used as drug pairs because of their similar effects. For example, the “Coptis chinensis-Sophora flavescens Tang” recorded in the “Puji Formula” includes 200 g of CC and 100 g of SF. In this prescription for the treatment of abdominal colic and high fever, CC is used as the primary drug and SF as an adjuvant drug [6,7]. The “Sophora flavescens pill” in “Jie Wei Yuan Suo” uses a combined use of SF and CC to treat itching in the back of the body [8]. Berberine Hydrochloride (BH) and Matrine (MT) are the main active ingredients of CC and SF, respectively (Figure 1). BH is an isoquinoline alkaloid compound with traditional antibacterial activity for the treatment of gastroenteritis and bacterial dysentery [9]. In recent years, it has been reported that BH can also play an anti-tumor role, inducing apoptosis in squamous cell carcinoma KYSE-70 cells, inhibiting the phosphorylation of Akt, mTOR, and p70S6K, and stimulating the activation of AMPK phosphorylation [10,11,12,13]. MT is a quinolizidine alkaloid with good pharmacological effects, such as anti-bacterial, anti-inflammatory, anti-viral, anti-fibrotic, anti-tumor, and anti-arrhythmic activities [14]. Modern pharmacological research has suggested that MT may exert anti-tumor effects by participating in autophagy and cell apoptosis. And it can activate many immune-related signaling pathways [15,16]. Inspired by the clinical use of CC and SF, we attempted to shift from the combined use of Chinese herbs to the combined use of natural compounds. The combination of natural compounds (BH and MT) can improve drug efficacy and reduce toxicity. In other words, we focused on the anti-tumor activity of BH as the main research objective and explored whether MT could serve as an adjuvant drug to enhance the anti-tumor activity of BH.

BH is a famous component of TCMs that has multiple pharmacological effects. However, the application of BH has been hampered by its poor hydrophobicity, stability, and bioavailability [17]. Liposomes are hollow macrovesicles formed by the encapsulation of lipid bilayers and are widely used in active and passive nanomedicine delivery systems because of their biodegradability, efficacy, and non-toxicity [18,19]. BH is slightly soluble in water and has no significant antibacterial or anti-tumor activity when used alone [20]. Therefore, modern drug delivery systems can convert BH into liposomes to solve the problem of poor water solubility [21]. In this research, a novel liposome was prepared by combining BH and MT to enhance the anti-tumor effect of BH. The anti-tumor effects of BH were investigated from two perspectives. First, we investigated whether the combination of BH and MT could enhance the anti-tumor effect of BH. The second aspect was to investigate whether liposomes could enhance the anti-tumor effects of BH. Two types of liposomes (BH-MT-LP; BH-LP) were successfully prepared by using the reverse evaporation method. The preparation process of BH-MT-LP and BH-LP was optimized by single-factor screening and the Box–Behnken experimental design (BBD) method, and the characteristics of the optimized liposomes were evaluated. Subsequently, the anti-tumor activity of four drugs (the unbound drugs BH, BH-MT, and the liposomes BH-LP; BH-MT-LP) was investigated. Finally, the anti-tumor activity of the unbound drug BH was used as a reference, and the anti-tumor activity of the other drugs (BH-MT, BH-LP, and BH-MT-LP) was preliminarily investigated. The anti-tumor activity of the combination of BH and MT was evaluated from multiple perspectives.

## 2. Results

### 2.1. The Results of Preparation of Liposomes

#### 2.1.1. Screening Results of Preparation Methods of BH-MT Liposomes

Liposome preparation methods were screened for encapsulation efficiency (EE) and drug loading (DL) as evaluation indicators. The EE and DL of BH-MT-LP prepared by the reverse evaporation method were the highest, whereas those of the liposomes prepared by the ammonium sulfate gradient method were the lowest (Table 1). Although the EE and DL of the other two methods were only slightly lower than those of the reverse evaporation method, there were some problems, such as residual organic solvent or excessive particle size. Therefore, after screening, the reverse evaporation method was used to prepare the BH-MT-LP.

#### 2.1.2. Single-Factor Screening Results of BH-MT-LP

##### The Effect of MT Content on EE and DL

**Figure 2:**
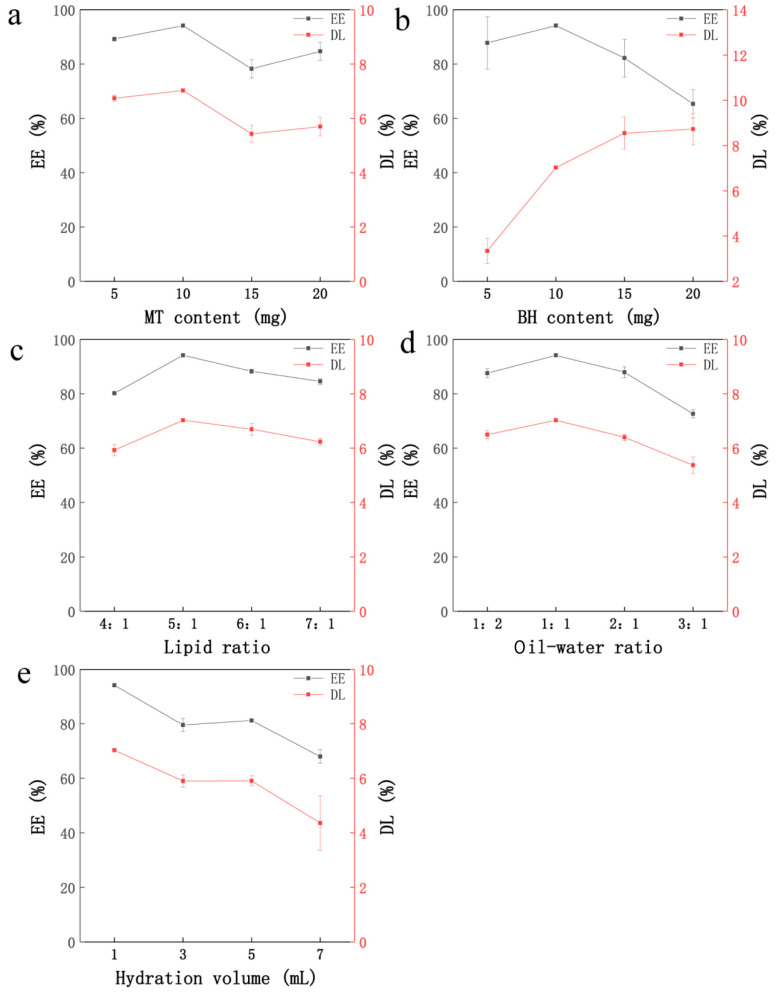
Single-factor screening results for the BH-MT-LP. MT content (**a**), BH content (**b**), lipid ratio (**c**), oil–water volume ratio (**d**), hydration volume (**e**).

The experiment showed that when the MT was 10 mg, the EE and DL of the liposomes were the best (Figure 2a). When the MT content was within the membrane saturation limit of the liposome, increasing the MT content may increase the EE and DL. However, when the MT exceeded 10 mg, it exceeded the membrane saturation limit of the liposomes. It leaked into the external water phase together with BH, which reduced the EE and DL of the liposomes. Therefore, MT (10 mg) was selected for follow-up experiments.

##### The Effect of BH Content on EE and DL

When the BH content was 10 mg, the EE of the liposomes was the highest, and when the BH content was 20 mg, the DL of the liposomes was the best (Figure 2b). During the preparation of the liposomes, with an increase in BH content, drug leakage may occur. And some of the unbound drug BH would adhere to the surface of the liposomes, resulting in lower EE and higher DL. For avoidance of the experimental error caused by this situation, BH (10 mg) with the highest EE was selected for the follow-up experiment.

##### The Effect of Lipid Ratio on EE and DL

Cholesterol can overregulate the extremely strong fluidity of phospholipids endowed with acyl chains, reduce the permeability of membranes, and increase EE and DL. If the cholesterol content exceeds the maximum load of the membrane, some liposomes are broken, resulting in a decrease in EE and DL [22,23]. Experimental data showed that when the lipid ratio was 5:1, the EE and DL of the liposomes were the highest (Figure 2c). As the proportion of lecithin in the mixture grew, the mobility within the lipid bilayer diminished, which in turn permitted the drug to escape into the surrounding watery environment. Consequently, the EE and DL of the liposomes experienced a continuous decline. Finally, we chose a lipid ratio of 5:1 for the subsequent experiments.

##### The Effect of Oil–Water Volume Ratio on EE and DL

At the oil–water volume ratio of 1:1, the EE and DL of the liposomes reached their peak values. However, as the oil–water volume ratio increased further, both EE and DL began to diminish (Figure 2d). The reason for this could be that increasing the amount of oil phase would lead to a decrease in the amount of encapsulated water-soluble drugs in the liposomes, which would affect the EE and DL of the liposomes. After comprehensive consideration, the oil–water ratio of 1:1 was selected for the experiment.

##### The Effect of Hydration Volume on EE and DL

The EE and DL of BH-MT-LP reached the maximum when the hydration volume was 1 mL (Figure 2e). The hydration volume was inversely proportional to the lipid concentration. Liposomes could not be formed if the hydration volume was too small, the lipid concentration was too high, and the viscosity was too high. If the hydration volume was too high and the lipid concentration too low, the drug cannot be encapsulated smoothly and the EE and DL would decrease. Therefore, the hydration volume of 1 mL was an appropriate experimental choice.

#### 2.1.3. Box–Behnken Experimental Design (BBD) Method Design and Results Analysis 

**Figure 3:**
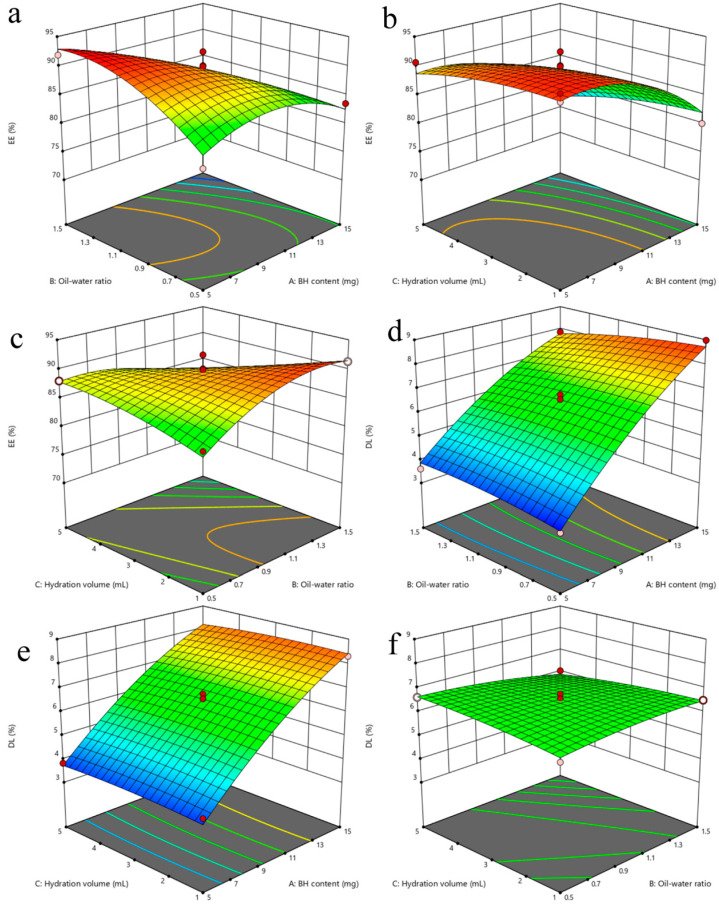
3D effect surface map of encapsulation efficiency and drug loading, where (**a**–**c**) are encapsulation efficiency 3D effect surface maps, and (**d**–**f**) are drug loading 3D effect surface maps. (In the graph, blue represents lower response values and red represents higher response values. Moreover, the center point of the smallest ellipse is usually the highest point on the BBD, which is the location of the optimal solution).

Based on single-factor experiments, it was found that BH content (A), oil–water ratio (B), and hydration volume (C) had rather significant influence on the EE and DL of the liposomes. So they were selected as the three evaluation factors for BBD. The relevant data for the optimization are shown in Table 2, Table 3 and Table 4, and the 3D response surface plots are shown in Figure 3. The results were analyzed by ANOVA using Design-Expert 12 software (the version number 12.0.3), and the regression equations of EE and DL were as follows:

R_EE_ = 89.58 − 5.32 A − 0.1662 B − 1.96 C − 4.84 AB − 0.4475 AC − 4.44 BC − 3.70 A^2^ − 2.97 B^2^ − 0.9485 C^2^ (R^2^ = 0.9316)

R_DL_ = 6.55 + 2.29 A − 0.135 B − 0.0525 C − 0.33 AB − 0.065 AC − 0.25 BC − 0.451 A^2^ − 0.136 B^2^ − 0.96 C^2^ (R^2^ = 0.9903)

The results of variance for the EE (Table 3) and DL (Table 4) showed the model *p* < 0.05 and the lack of fit item *p* > 0.05, indicating that the model can be used for optimal process prediction and was applicable. The analysis of variance shown in Table 3 showed that factors A, C, AB, BC, A^2^, and B^2^ had a significant impact on EE (*p* < 0.05). The analysis of variance shown in Table 4 showed that factors A, AB, and A2 significantly influenced DL (*p* < 0.05).

Quadratic equations and 3D response surface morphology reflect the influence of independent variables on liposome EE and DL. In the 3D response surface plot, the change in color and slope reflects the influence of the independent factors on the EE and DL. In general, the darker the color and the greater the slope, the greater the influence [24,25]. The regression coefficients of the EE response surface indicated that EE was influenced by the negative effects of factors A, B, and C, with C having the greatest effect. Increasing factor C led to a decrease in phospholipid concentration, resulting in a decrease in drug EE. The effect surface analysis showed that, as factor A increased, EE first increased and then decreased (Figure 3). In addition, an appropriate increase in factor A promoted improvement in EE. However, owing to the limited ability of a certain amount of lipids to contain encapsulated compounds, once the encapsulation saturation of drug liposomes was exceeded, EE decreased with increasing BH addition. The regression coefficients of the DL response surface indicated that factor A had a significant positive impact on DL, whereas factors B and C had a relatively small negative impact on DL. Therefore, DL increased with the increase in factor A, and the 3D response surface containing A exhibited a shape similar to that of an inclined plane, whereas the 3D response surface formed by B and C had a planar shape.

Based on the results of the BBD experiments, Design-Expert 12 software predicted the optimal process: 11 mg BH, an oil–water ratio of approximately 6:5, and a hydration volume of 1 mL. To verify the reliability of the model equation, three batches of liposomes were prepared according to the predicted optimal process, and EE and DL were measured. Meanwhile, EE reached a forecast of 89.97% and DL reached 7.01%. As shown in Table 5, the deviations between the measured and predicted values of EE and DL were −0.0084% and 0.0028%, respectively, which are in agreement with the predicted values. The results of the binomial fitting were good and highly reliable.

### 2.2. Characterization of BH-MT-LP

#### 2.2.1. Appearance Form

**Figure 4:**
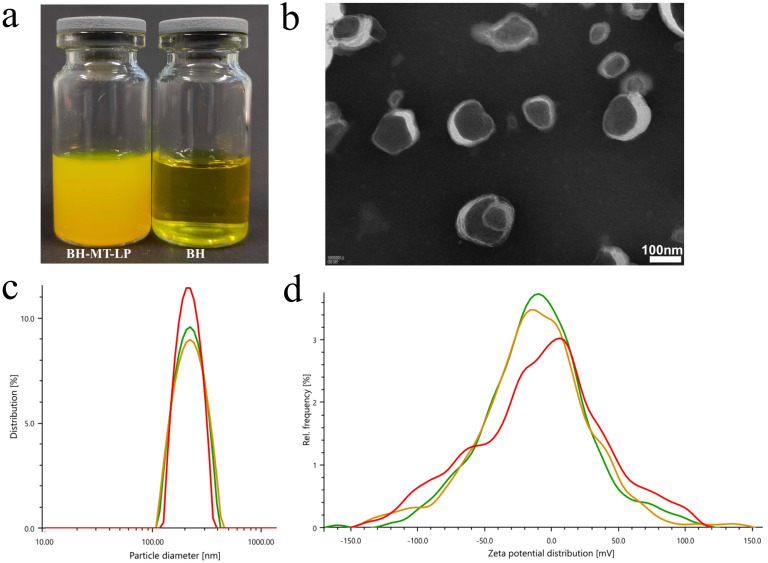
Characterization of BH-MT-LP. (**a**) appearance; (**b**) electron microscope; (**c**) particle size; (**d**) zeta potential. In (**c**,**d**), the lines of varying colors correspond to the three times of measurements conducted.

Under optimum conditions, the BH-MT-LP prepared by the reverse evaporation method was a yellow suspension. The liposomes appeared as a pale yellow milky light when illuminated after dilution 5-fold (Figure 4a).

#### 2.2.2. Morphology Analysis Using Transmission Electron Microscope

As shown in Figure 4b, the liposomes were spherical, as observed under a transmission electron microscope.

#### 2.2.3. Particle Size and Potential

The particle size of the liposome measured by the Malvin particle size analyzer ranged from 222.7 nm to 235.4 nm, and the polydispersity index (PDI) ranged from 11.8% to 23.3%. The zeta potential was −35.9~−31.1 mV (Figure 4c,d).

### 2.3. In Vitro Release of BH-MT-LP

**Figure 5:**
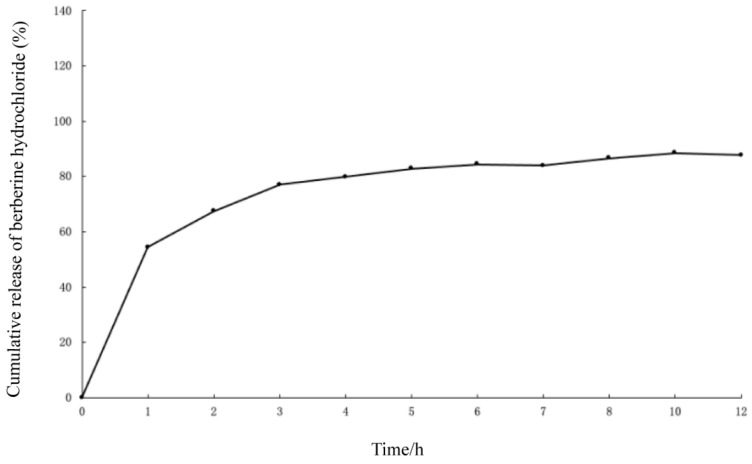
Cumulative release curve of BH in BH-MT-LP.

The process of liposome release in vitro can be divided into two stages: burst release and slow release. The rapid release in the burst release stage is mainly because it is an unencapsulated free drug. However, the slow release in the sustained-release stage is mainly because the drugs encapsulated in liposomes need to cross the phospholipid bilayer, thus having a sustained release and playing a long-lasting role [26]. From the cumulative release curve, it can be seen that the BH in the liposomes reached 80% within 5 h (Figure 5). BH-MT-LP had a slow release rate because some of the drugs were encapsulated in the liposomes.

### 2.4. Anti-Tumor Results 

#### 2.4.1. Cytotoxicity of BH-MT-LP in Different Cell Lines

**Figure 6:**
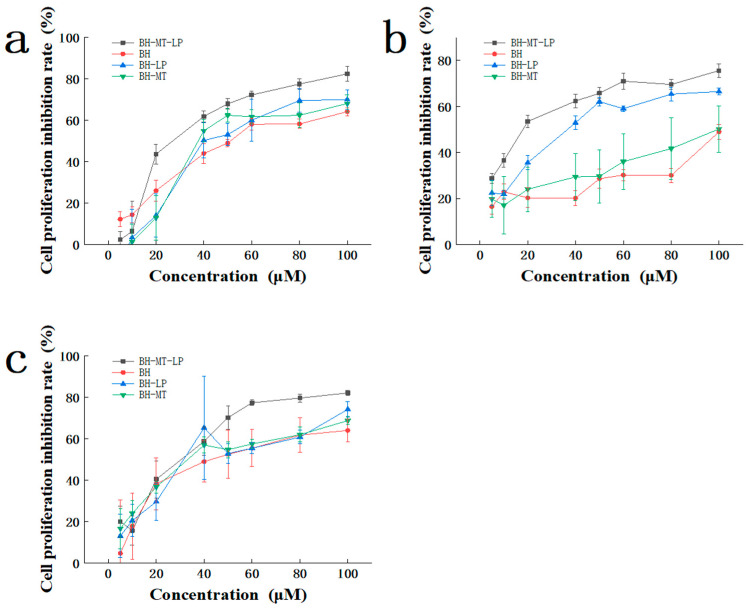
Cytotoxicity of BH-MT-LP, BH, BH-LP and BH-MT in different cell lines. MDA-MB-231 (**a**), HepG-2 (**b**), HGC-27 (**c**).

In vitro cytotoxicity testing is an important index for evaluating the properties of liposomes. It includes information such as whether drugs can be ingested by cells and whether drugs can be effectively released [27,28]. Therefore, we used the MTT assay to determine the cytotoxicity of four drugs (BH-MT-LP, BH-LP, BH-MT, and BH) at different concentrations in three cell lines. These three cell lines are a human breast cancer cell line (MDA-MB-231), human liver cancer cell line (HepG-2), and human gastric cancer cell line (HGC-27). With an increase in drug concentration, the proliferation inhibition rates of the four drugs in the MDA-MB-231, HepG-2, and HGC-27 cell lines also increased. All the drugs exhibited significant cytotoxicity and inhibited tumor cell growth in a dose-dependent manner (Figure 6a–c).

In the MDA-MB-231 cell lines, across concentrations from 20 to 100 µM, BH-MT-LP exhibited a more potent inhibitory effect than BH, BH-LP, and BH-MT, indicating significant anti-tumor activity (Figure 6a). Within the concentration range of 40 to 100 µM, the drug BH-MT showed a higher inhibition rate against tumor cells compared to the single drug BH (BH-MT > BH). Similarly, the liposome BH-MT-LP showed superior inhibitory effects compared to BH-LP (BH-MT-LP > BH-LP). The liposomes BH-MT-LP and BH-LP demonstrated higher inhibition rates compared to their unbound drugs, BH-MT and BH, respectively (BH-MT-LP > BH-MT; BH-LP > BH). Moreover, in the HepG-2 cell lines, this effect was more pronounced (Figure 6b). When the drug concentration was in the range of 20 to 100 µM, BH-MT showed a superior inhibition rate against HepG-2 cells compared to the single drug BH (BH-MT > BH). And the inhibition rate of BH-MT-LP was also better than that of BH-LP (BH-MT-LP > BH-LP). The anti-tumor activity of the liposomes (BH-MT-LP and BH-LP) was much greater than that of the unbound drugs (BH-MT and BH). Nevertheless, the impact of the drugs on the HGC-27 cell line was not as pronounced as it was for the MDA-MB-231 and HepG-2 cell lines (Figure 6c). Within the concentration range of 50–100 µM, BH-MT exhibited a higher inhibition rate compared to BH, and the liposome BH-MT-LP showed a more pronounced effect than BH-LP. Moreover, the efficacy of BH-MT-LP surpassed that of BH-MT, while BH-LP demonstrated a slightly higher efficacy than BH.

Upon evaluating the cytotoxicity of the four drugs against the three distinct cell lines, it was observed that the combination of two drugs yielded a more potent anti-tumor effect than the use of a single drug (BH-MT > BH; BH-MT-LP > BH-LP). Furthermore, the anti-tumor efficacy of the drugs when encapsulated in liposomes was found to be superior to that of the unbound drugs (BH-MT-LP > BH-MT; BH-LP > BH). It is evident that BH-MT-LP exhibits a higher rate of cell inhibition among the four drugs when assessing their anti-tumor activities. In summary, BH-MT-LP displays varying effects across different cell lines. The encapsulation of BH and MT within liposomes not only enhances their therapeutic efficacy but also broadens their potential applications to combat breast cancer, liver cancer, and gastric cancer, thereby expanding their spectrum of anti-tumor activity.

#### 2.4.2. The Effect of BH-MT-LP on Cell Apoptosis

**Figure 7:**
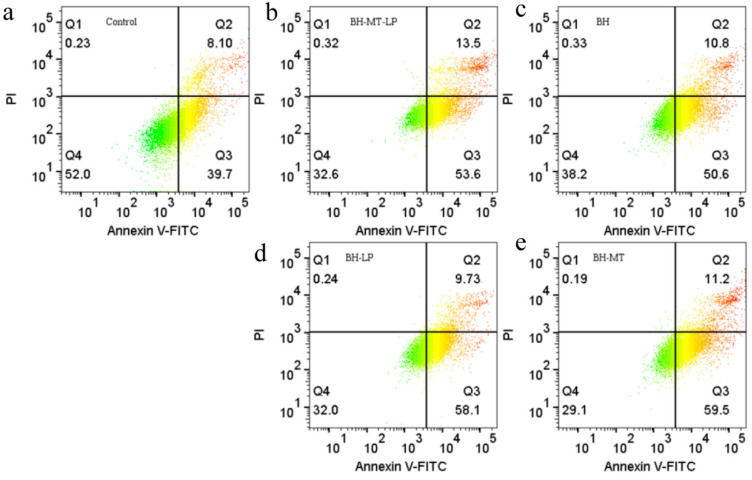
Cell apoptosis of MDA-MB-231 cells after treatment with BH-MT-LP, BH, BH-LP, and BH-MT for 48 h. Control group (**a**), BH-MT-LP group (**b**), BH group (**c**), BH-LP group (**d**), BH-MT group (**e**).

Based on the toxicity of BH-MT-LP in MDA-MB-231 cells, we continued to investigate the molecular basis of the inhibitory mechanism of BH-MT-LP on cell growth. Annexin V/PI double staining was used to quantify the degree of apoptosis after 48 h of drug treatment. The four quadrants represent live cells (Q4), early apoptotic cells (Q3), late apoptotic cells (Q2), and necrotic cells (Q1), respectively (Figure 7a). Different dosages result in significant differences in cell populations at different stages of death. After BH treatment, the early and late apoptotic cell populations (Q3 and Q2 phases) increased to 50.6% and 10.8% (Figure 7c), while BH-MT treatment increased them to 59.5% and 11.2% (Figure 7e), BH-LP treatment increased them to 58.1% and 9.73% (Figure 7d), and BH-MT-LP treatment increased them to 53.6% and 13.5% (Figure 7b), respectively. Compared with the control group, the percentage of live cells decreased significantly after drug treatment and the number of early and late apoptotic cells increased. The results indicated that all four drugs can promote the apoptosis of tumor cells. The data showed that the total apoptosis rate in the groups treated with the combination therapies (BH-MT-LP and BH-MT) was substantially higher compared to the group treated with the single drug BH, suggesting that the co-administration of BH and MT potentiated the apoptotic effect on tumor cells.

Compared with the single drug BH, the total apoptosis rate of the liposome group (BH-MT-LP and BH-LP) was significantly increased, indicating that liposomes can enhance the apoptotic effect of the drug on tumor cells and exhibit effective anti-tumor effects. In addition, BH-MT-LP showed a more pronounced apoptotic effect in the late stage of apoptosis, whereas BH-MT showed a more pronounced apoptotic effect in the early stage of apoptosis. These quantitative data analyses indicated that the combined use of BH and MT with liposome technology was more effective in the promotion of tumor cell apoptosis. BH-MT-LP increased the apoptosis rate of BH in MDA-MB-231 and significantly promoted its apoptosis, which was consistent with the cytotoxicity results. 

#### 2.4.3. The Effect of BH-MT-LP on Cell Cycle

**Figure 8:**
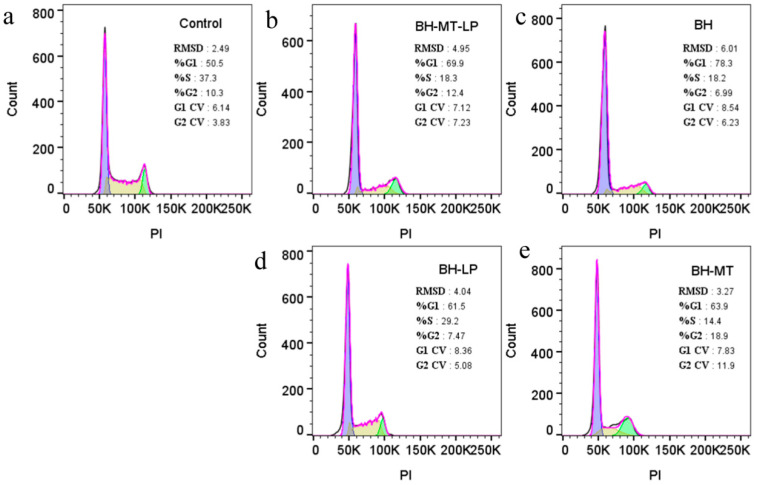
Cell cycle of MDA-MB-231 cells after treatment with BH-MT-LP, BH, BH-LP, and BH-MT at 50 µM for 48 h. Control group (**a**), BH-MT-LP group (**b**), BH group (**c**), BH-LP group (**d**), BH-MT group (**e**).

The cell cycle refers to the entire process that a cell undergoes from the completion of one division to the end of the next, divided into two phases: interphase and division. Among them, the interphase is divided into three phases, namely the pre-DNA phase (G1 phase), the DNA synthesis phase (S phase), and the post-DNA synthesis phase (G2 phase), which provide material for cell proliferation. Cell cycle arrest is considered a key factor in preventing malignant tumors [29,30]. To investigate whether the proliferation inhibition of the MDA-MB-231 cell proliferation following treatment was due to cell cycle arrest, we analyzed the data of PI-stained cells by flow cytometry. For the G1 phase, the percentage of cells in the treatment groups was higher than that of the control group (50.5%) (Figure 8a). The BH group showed an increase to 78.3% (Figure 8c), the BH-MT group to 63.9% (Figure 8e), the BH-LP group to 61.5% (Figure 8d), and the BH-MT-LP group to 69.9% (Figure 8b). In the S phase, the proportion of cells was significantly lower in the treatment groups compared to the control cells (37.3%) (Figure 8a). The BH group showed a reduction to 18.2% (Figure 8c), the BH-MT group to 14.4% (Figure 8e), the BH-LP group to 29.2% (Figure 8d), and the BH-MT-LP group to 18.3% (Figure 8b). Compared with the control group, all four drugs increased the number of G1-phase cells and decreased the number of S-phase cells in each experimental group. In other words, under drug treatment, the MDA-MB-231 cancer cells showed significant cell aggregation in the G1 phase, while the number of cells in the S phase decreased significantly. This suggested that the drug mainly affected the G1 and S phases of the cells, increasing cell apoptosis.

## 3. Discussion

This study demonstrated from two perspectives that the combination of medicinal herbs can be transformed into the combination of the main active ingredients, proving this approach viable. Concurrently, this research also showed that the combination of BH and MT, as well as liposome encapsulation, can enhance the anti-tumor effect of BH. The single compound BH and the combined use drug BH-MT were encapsulated in liposomes. We have successfully prepared two types of BH-containing liposomes (BH-LP; BH-MT-LP) and optimized the process. The anti-tumor activity of the four drugs (BH, BH-MT, BH-LP, and BH-MT-LP) were investigated with the following conclusions.

### 3.1. BH-MT-LP Process Method Screening and Optimization 

BH’s clinical application is significantly hindered by its poor water solubility, low absorption rates, intense first-pass metabolism, and inadequate bioavailability [31]. The encapsulation of BH in liposomes can improve the bioavailability of the drug and achieve a slow-release effect [17,32,33]. Generally, methods for the preparation of liposomes include the thin-film dispersion method, reverse evaporation method, ether injection method, and ammonium sulfate gradient method [34]. After screening four methods by EE and DL as evaluation criteria, the reverse evaporation method was ultimately chosen for the liposome preparation. The optimal preparation process was optimized through the single-factor screening method and the BBD method. Then, two liposomes (BH-MT-LP and BH-LP) with excellent stability, small nanoparticle size, and stable zeta potential were successfully prepared. At the same time, in vitro release experiments showed that BH in BH-MT-LP released slowly with a cumulative release rate of 80%. 

### 3.2. Results of Anti-Tumor Experiments

Previous studies have confirmed that BH possesses therapeutic efficacy against a range of cancers, such as liver, breast, and stomach cancers [35,36,37,38]. BH has been proven to affect the cell cycle, apoptosis, autophagy, and tumor microenvironment [36]. Furthermore, MT has also demonstrated positive therapeutic effects in the treatment of breast, liver, and gastric cancers [39,40,41,42]. Both ancient texts and modern pharmacological studies have shown that the pharmacological effects of BH and MT are similar and that the combination of the two components is reasonable [7,43,44,45]. In this context, this study demonstrated that the combined use of BH and MT could enhance the anti-tumor effect of BH. Moreover, the encapsulation of BH in liposomes, which provides a sustained release, further amplified the anti-tumor effect of BH.

#### 3.2.1. Cytotoxicity Experiments

Based on the literature, we selected three cell lines for the vitro toxicity testing [46]. The findings showed that these four drugs (BH, BH-MT, BH-LP, and BH-MT-LP) had inhibitory effects on the MDA-MB-231, HepG-2 and HGC-27 cell lines and inhibited tumor cell formation in a dose-dependent manner. From Figure 6, it can be observed that the anti-tumor effect of BH-MT was higher than that of BH in the unbound drug group (BH-MT > BH), and it can also be demonstrated from the liposome group that the anti-tumor effect of BH-MT-LP was higher than that of BH-LP (BH-MT-LP > BH-LP). Comparing the cytotoxicity of four drugs on three different cell lines, it was found that the combined use of two drugs exerted a stronger anti-tumor effect than the single drug (BH-MT > BH; BH-MT-LP > BH-LP). This showed that the combined use of BH and MT could enhance the drug effect. Furthermore, analyzing Figure 6 from a different perspective showed that BH-MT-LP had a stronger anti-tumor effect than BH-MT (BH-MT-LP > BH-MT), and BH-LP had a stronger anti-tumor effect than BH (BH-LP > BH). The liposomal group was more potent than the unbound drug group (BH-MT-LP > BH-MT; BH-LP > BH). We speculated that the sustained release of the liposomes may be able to stabilize the release of the BH and improve its therapeutic efficacy. Upon close examination, it is evident that BH-MT-LP exhibited a higher rate of cell inhibition among the four drugs when evaluating their anti-tumor activities. These results indicated that we have successfully prepared a BH-containing liposome with strong anti-tumor activity. Currently, studies have indicated that the synergistic use of BH and MT can boost therapeutic outcomes, primarily in the context of antibacterial treatments, with no clear evidence yet of its impact on anti-tumor therapies [47,48]. Additionally, the majority of liposomal formulations reported to date encapsulate either BH or MT, with no studies identified that explore the co-encapsulation of both drugs within liposomes [49,50]. Hence, the BH-MT-LP developed in this study holds significant implications for enhancing the anti-tumor efficacy of BH.

#### 3.2.2. Cell Apoptosis Experiments

BH demonstrates anti-neoplastic effects across a spectrum of cancers, with potential mechanisms including the suppression of cancer cell growth and the promotion of cell apoptosis [51]. Based on the toxicity of BH-MT-LP in MDA-MB-231 cells, we continued to investigate the inhibitory effect of BH-MT-LP on cell growth. Figure 7 showed that after drug treatment, the total apoptosis rate of the four drugs (BH, BH-MT, BH-LP, and BH-MT-LP) was significantly increased compared to the control group (47.8%). It was indicated that all four drugs promoted the apoptosis of tumor cells. From Figure 7, it can be seen that the total apoptosis rate of the BH-MT, BH-LP, and BH-MT-LP groups was higher than that of the BH group (61.4%). The total apoptosis rate of the combined use treatment groups (BH-MT-LP and BH-MT) was significantly higher than that of the single drug BH, indicating that the combined use of BH and MT enhanced the apoptosis of tumor cells. These experimental results confirmed that the combined use of BH and MT could enhance therapeutic effects. Compared with the single drug BH, the total apoptosis rate of the liposome groups (BH-MT-LP and BH-LP) was significantly increased, indicating that liposomes enhanced the apoptotic effect of the drug on tumor cells. The mechanism of action may be that the liposomes increased the stability of the BH so that it had a sustained effect on the tumor cells. Additionally, BH-MT-LP showed a more pronounced apoptotic effect in the late stage of apoptosis, whereas BH-MT showed a more pronounced apoptotic effect in the early stage of apoptosis. This result fully proved that the liposome could achieve a slow release of BH-MT. In the cell apoptosis experiment, it was not only shown that the addition of MT improved the anti-tumor effect of BH but also that the sustained release of liposomes could enhance the anti-tumor effect of BH.

#### 3.2.3. Cell Cycle Experiments

Regarding the induction of cell cycle arrest, BH has cytotoxic effects on several cancer cell lines [52]. The data in Figure 8 showed that all four drugs (BH, BH-MT, BH-LP, and BH-MT-LP) increased the number of G1-phase cells and decreased the number of S-phase cells in each experimental group compared to the control group (G1 phase 50.5%; S phase 37.3%). In other words, under drug treatment, the MDA-MB-231 cancer cells showed significant cell aggregation in the G1 phase, while the number of cells in the S phase decreased significantly. These four drugs showed similar trends in their effects on the tumor cell cycle. Figure 8 indicates that a higher percentage of S-phase tumor cells was observed with BH-LP (29.2%) than with BH (18.2%), suggesting that liposomal encapsulation improved the sustained-release profile of BH. Likewise, the percentage of S-phase tumor cells influenced by BH-MT-LP (18.3%) was markedly greater compared to those affected by BH-MT (14.4%). Overall, BH-MT-LP can promote the apoptosis of tumor cells by blocking the cell cycle (G1 and S phase). Moreover, the liposomal formulation provides a sustained-release effect, which extends the duration of drug release and enhances bioavailability.

## 4. Materials and Methods

### 4.1. Materials and Cell Lines 

Berberine hydrochloride (BH, PS020505, purity > 98%, Chengdu Pusi Biotechnology Co., Ltd., Chengdu, China), Marine (MT, PS011495, purity > 98%, Chengdu Pusi Biotechnology Co., Ltd., Chengdu, China), soybean lecithin (2021050601, Chengdu Cologne Chemicals Co., Ltd., Chengdu, China), cholesterol (2020010101, Chengdu Cologne Chemicals Co., Ltd., Chengdu, China), anhydrous ether (2109087, Sichuan Xilong Science Co., Ltd., Chengdu, China), potassium dihydrogen phosphate (2018062201, Sichuan Xilong Science Co., Ltd., Chengdu, China), and latong X-100 (RH350084, Shanghai Linen Technology Development Co., Ltd., Shanghai, China) were obtained. Acetonitrile and methanol (HPLC grade) were obtained from Thermo Fisher Scientific Shier (Beijing, China) Technology Co., Ltd. All other reagents were analytical grade and purified deionized water was used throughout.

The human gastric cancer cell line (HGC-27) came from Wuhan Punuosai Biotechnology Co., Ltd., (Wuhan, China). The human breast cancer cell line (MDA-MB-231) and human liver cancer cell line (HepG-2) were donated by Southwest State Key Laboratory of Traditional Chinese Medicine Resources.

### 4.2. Preparation of Liposomes

BH is slightly soluble in water; usually 1 g can be dissolved in 10–100 mL of water. And MT can be dissolved in water; usually 1 g can be dissolved in 10–30 mL of water [5,53]. In this experiment, 10 mg of BH and MT were dissolved in 10 mL of pure water to make a 1 mg/mL drug solution. In addition, through a preliminary literature review, the preparation process of the liposomes was preliminarily determined with a preparation temperature of 50 °C, solvent as anhydrous ether, and lipid ratio of 5:1 (100 mg lecithin and 20 mg cholesterol) [54,55,56].

#### 4.2.1. Determination Method for EE and DL

##### Determination of BH Content by HPLC Method

The BH content was detected (HPLC-Angilent 1260 Infinity II) at 345 nm using a reversed-phase column (Agilent C18, 250 mm × 4.6 mm, 5 um). The mobile phase was a mixture of acetonitrile (B) −0.01 mol buffer stock aqueous solution (B) at a flow rate of 1 mL/min and an injection volume of 20 µL (Table 6) [5]. The concentration of BH was calculated using the following standard curve: y = 59658x − 25.359 (R^2^ = 0.9996). The standard curve was constructed using a series of standard BH solutions with concentrations ranging from 0.992 µg/mL to 49.6 µg/mL. 

##### Determination of Free Drug Content

To determine the free drug content, first, 1 mL of the liposomes were accurately aspirated and loaded into a pre-treated dialysis bag with a molecular cut-off of 10,000. The dialysis bag was then placed in a beaker containing 100 mL of pure water and dialyzed for 5 h. The dialysate was filtered through a 0.22 µm microporous membrane and the peak area (A) of BH at a wavelength of 345 nm was measured by HPLC. We calculated the free BH content after 5 h of dialysis (W2).

##### Total Drug Content Determination

The non-dialyzed 1 mL liposomes were precisely measured in a 10 mL volumetric bottle, followed by the addition of 3 mL of 10% TX-100 emulsion breaker. The pure water solution was diluted to volume and shaken well. Next, 1 mL of the solution was transferred to a 10 mL volumetric flask. After dilution with pure water, an appropriate volume was taken and filtered through a 0.22 µm (Tianjin Jinteng Experimental Equipment Co., Ltd., Tianjin, China) microporous membrane. The filtrate was transferred to an injection vial and the peak area of BH was determined by HPLC at a wavelength of 345 nm. The total BH content in 1 mL of liposomes (W1) was calculated.

EE% = (W1 − W2)/W1 × 100%

DL% = (W1 − W2)/W × 100%

(W1 was the total content of BH in liposomes, W2 was the content of free BH in liposomes, and W was the total content of lecithin, cholesterol, MT, and BH).

#### 4.2.2. Screening Results of Preparation Methods of BH-MT-LP

##### Thin-Film Dispersion Method

First, 100 mg lecithin and 20 mg cholesterol were precisely weighed in a pear-shaped bottle and dissolved in 10 mL of anhydrous ether. Subsequently, the mixed solution was placed on a rotary evaporator (RE-52AA, Shang Hai Yarong Biochemistry Instrument Factory, Shanghai, China), and the anhydrous ether was removed under a vacuum at 30 °C. When a uniform film was formed on the bottle, a 10 mL water solution of BH and MT (1 mg/mL) was added. The film was then hydrated at 50 °C for 1 h. The liposome suspension was ultrasonicated for 10 min for uniform dispersion. Finally, the liposome suspension was filtered through 0.45 μm and 0.22 μm microporous membranes (Tianjin Jinteng Experimental Equipment Co., Ltd., Tianjin, China) [57].

##### Reverse Evaporation Method

Lecithin (100 mg) and cholesterol (20 mg) were precisely measured in a pear-shaped flask and dissolved in 10 mL of anhydrous ether. Concurrently, BH (10 mg) and MT (10 mg) were precisely weighed into a separate pear-shaped flask and dissolved in 10 mL of ultrapure water. The drug solution (1 mg/mL) was injected uniformly into an anhydrous ether solution. After ultrasonic treatment for 10 min, an emulsion was formed in which the oil phase enveloped the water phase (W/O emulsion). Subsequently, the emulsion was placed on a rotary evaporator, and the organic solvent was removed under a vacuum at 30 °C. After removing all the anhydrous ether, 20 mL of ultrapure water solution was added. The yellow suspended liposomes were obtained by hydration for 1.0 h at 50 °C. They were filtered through 0.45 μm and 0.22 μm microporous membranes to obtain the BH-MT-LP [58].

##### Ether Injection Method

Lecithin (100 mg) and cholesterol (20 mg) were precisely measured in a pear-shaped flask and dissolved in 10 mL of anhydrous ether. Concurrently, BH (10 mg) and MT (10 mg) were precisely weighed into a separate pear-shaped flask and dissolved in 10 mL of ultrapure water. The ether solution was set on a magnetic stirrer at a constant temperature of 40 °C and stirred at a speed of 100 rpm. The drug solution (1 mg/mL) was then slowly injected. The solution was stirred for 1 h until all anhydrous ether was removed. Thus, yellow suspended liposomes were obtained and filtered through 0.45 μm and 0.22 μm microporous membranes to obtain the BH-MT-LP [56].

##### Ammonium Sulfate Gradient Method

Firstly, lecithin (100 mg) and cholesterol (20 mg) were precisely measured in a pear-shaped flask and dissolved in 10 mL of anhydrous ether. Concurrently, BH (10 mg) and MT (10 mg) were precisely weighed into a separate pear-shaped flask and dissolved in 10 mL of ultrapure water. Subsequently, the ether solution was placed on a rotary evaporator, and the anhydrous ether was removed under a vacuum at 30 °C. When a uniform film was formed on the bottle, a 5 mL 0.3 mol/L ammonium sulfate solution was added. The film was then hydrated at 50 °C for 1 h. Secondly, the liposome suspension was ultrasonicated for 10 min for uniform dispersion. It was then placed on a magnetic stirrer at a constant temperature of 50 °C and stirred for 10.0 min before being converted to empty liposomes. Finally, the empty liposomes were enclosed in a dialysis bag with a molecular weight cut-off of 10,000 Da. The dialysis bag was immersed in 500 mL of pure water for a 24 h dialysis period. After dialysis, 10 mL of drug solution (1 mg/mL) was injected into the empty liposomes. Then the liposomes were incubated in a magnetic stirrer at a constant temperature of 50 °C for 1 h. The BH-MT-LP was obtained by filtering through 0.45 μm and 0.22 μm microporous membranes [59,60,61].

#### 4.2.3. Single-Factor Screening Experiment of BH-MT-LP

The EE and DL of BH-MT-LP were affected by numerous factors throughout the liposome preparation process. This study employed a single-factor screening experiment to assess the impact of various factors on the EE and DL of BH-MT-LP, with the goal of identifying the optimal preparation technique.

##### The Effect of MT Content on EE and DL

The lipid ratio (5:1), BH (10 mg), oil–water volume ratio (1:1), and hydration volume (1 mL) in the prescription were kept constant, while the content of MT (5, 10, 15, 20 mg) was varied. The optimal MT content was selected for subsequent experiments based on the EE and DL of the liposomes as evaluation indicators.

##### The Effect of BH Content on EE and DL

In the formulation, the lipid ratio (5:1), MT (10 mg), oil–water volume ratio (1:1), and hydration volume (1 mL) were held constant, and the content of BH was varied at levels of 5, 10, 15, and 20 mg. The optimal BH content was screened for further experiments based on the EE and DL of the liposomes, which served as the key evaluation metrics.

##### The Effect of Lipid Ratio on EE and DL

The quantities of BH at 10 mg, MT at 10 mg, the oil-to-water volume ratio at 1:1, and the hydration volume at 1 mL in the formulation were kept constant. The study then explored different lipid ratios (4:1, 5:1, 6:1, 7:1) to determine their impact. The most suitable lipid ratio was selected for further experimentation, with the EE and DL of the liposomes serving as the primary criteria for evaluation.

##### The Effect of Oil–Water Volume Ratio on EE and DL

The fixed parameters in the formulation included BH at 10 mg, MT at 10 mg, a lipid ratio of 5:1, and a hydration volume of 1 mL. The study investigated varying oil–water volume ratios, which were 1:2, 1:1, 2:1, 3:1, and 4:1. The most effective oil–water volume ratio was determined for subsequent experiments based on the EE and DL of the liposomes.

##### The Effect of Hydration Volume on EE and DL

The composition of the formulation, with a lipid ratio of 5:1, BH at 10 mg, MT at 10 mg, and an oil-to-water volume ratio of 1:1, remained constant. The study varied the hydration volume, examining 1 mL, 3 mL, 5 mL, and 7 mL, to determine its optimal level. The selection of the ideal hydration volume for further experiments was based on the EE and DL.

#### 4.2.4. BBD Study

Uniform and orthogonal designs are frequently utilized in pharmaceutical development and prescription screening due to their small number of tests and straightforward data analysis [62]. However, these methods suffer from limited accuracy as they often overlook the potential interactions among various factors [63,64]. In contrast, the BBD method is a multifactorial, nonlinear testing optimization technique that requires fewer trials than orthogonal design method, yet offers greater precision. It stands out for its simplicity, comprehensiveness, and suitability for multifactorial testing [65]. According to the single-factor screening experiments, the content of BH (A), the oil–water ratio (B), and hydration volume (C) were selected as key factors. The EE and DL of BH-MT-LP served as the evaluation criteria. The BBD method was then employed to optimize the liposome preparation process.

#### 4.2.5. Characterization of BH-MT-LP

##### Appearance Form

We accurately measured 10 mL of BH-MT-LP and observed its color and condition. Simultaneously, the liposome was diluted 5-fold and its color and condition were observed under the light.

##### Morphology of Transmission Electron Microscope

The structure of BH-MT-LP was examined using transmission electron microscopy [56]. We accurately measured 0.2 mL of BH-MT-LP and diluted it 15-fold, and a 10 µL aliquot was placed onto a special copper mesh. When the liquid had evaporated naturally, 10 µL of liposomes were added and the procedure was repeated twice. The liposome particles were concentrated and deposited online. Then, 1% phosphotungstic acid solution was added dropwise for negative staining. After allowing the sample to air dry, the morphology of the liposomes was visualized under transmission electron microscopy.

##### Particle Size and Potential

A 1 mL sample of BH-MT-LP was diluted 20-fold with physiological saline and placed in the sample cell of the Malvin particle size analyzer [66]. The analyzer was then used to determine both the particle size and the zeta potential of the liposomes.

##### In Vitro Release of BH-MT-LP

A precise 1 mL of BH-MT-LP was measured and introduced in a dialysis bag, which was then submerged in 100 mL of pure water for the dialysis process. At intervals of 0, 1, 2, 3, 4, 5, 6, 7, 8, 10, and 12 h, 1 mL of the dialysate was collected, and an equivalent volume of water was added to the dialysis bag to maintain the total volume. The cumulative release curve was constructed with the dialysis time plotted on the *x*-axis and the concentration of BH in the dialysis on the *y*-axis.

The viability of the cells in each well was assessed using the MTT assay, the data were documented, and the inhibition rate of cell proliferation was calculated [67,68,69,70,71].

### 4.3. Anti-Tumor Experiments

#### 4.3.1. Cytotoxicity Test

MDA-MB-231, HepG2, and HGC-27 cells, which were in the logarithmic growth phase and growing well, were evenly seeded in a 96-well plate at a density of 5×10^3^ cells per well. When the cells had fully adhered to the wall, the culture medium in the well was discarded, and 100 μL of culture medium (10% FBS and 1% penicillin–streptomycin) containing liquid drug was added to each well. The study was organized into four groups (BH-MT-LP, BH-LP, BH, and BH-MT), with each group featuring eight different concentrations (5, 10, 20, 40, 50, 60, 80, 100 μM) and triplicate wells for each concentration. After a 48 h incubation period, the medium containing the drug was aspirated. The viability of the cells in each well was assessed using the MTT assay, the data were documented, and the inhibition rate of cell proliferation was calculated [67,68,69,70,71].

$$\;\mathrm{Cell}\;\mathrm{proliferation}\;\mathrm{inhibition}\;\mathrm{rate}=\frac{1-Experimental\;group\;A\;values}{Blank\;Group\;A\;Value}\times 100\%$$

#### 4.3.2. Determination of Cell Apoptosis

Well-growing MDA-MB-231 cells in the logarithmic growth phase were seeded uniformly in a 12-well plate at a concentration of 1 × 10^5^ cells per well. Following a 24 h incubation period, 50 μM concentrations of various drugs (BH-MT-LP, BH-LP, BH, BH-MT) were added to each well, with a control group receiving no treatment. After an additional 48 h of cultivation, adherent and suspended cells were collected in centrifuge tubes. The cells were centrifuged at 2000 rpm for 5 min, the supernatant was removed, and the cells were washed twice with cold PBS. Subsequently, the cells were stained with 5 µL of Annexin V-FITC and 5 µL of propidium iodide, and after a 10–20 min incubation at room temperature in the dark, flow cytometry analysis was conducted [72].

#### 4.3.3. Cell Cycle Detection

MDA-MB-231 cells, which were in good health and at the logarithmic growth phase, were evenly seeded in a 12-well plate at a rate of 1 × 10^5^ cells per well. After 24 h of incubation, 50μM of the test drugs (BH-MT-LP, BH-LP, BH, BH-MT) were introduced into each well, while the control group received no treatment. Then, after 48 h of further cultivation, both attached and non-attached cells were gathered into centrifuge tubes. The tubes were centrifuged, after which the supernatant was removed and the cells were washed twice using chilled PBS. The cells were then treated with 1 mL DNA staining solution and 10 µL permeabilization solution. The staining solution was briefly vortexed for 5–10 s and left to incubate in the dark at room temperature for 30 min. Finally, the stained cells were analyzed using flow cytometry [73].

### 4.4. Statistical Analysis

Experimental data were expressed as mean ± standard deviation (SD) and all experiments were performed at least three times in parallel. An unpaired *t*-test was performed on the data using SPSS 26.0 software, and *p* < 0.05 was considered statistically significant.

## 5. Conclusions

This trial successfully produced BH-MT-LP with desirable stability, small particle size, and a stable zeta potential through process optimizing and fine-tuning. The release rate of the drug from the liposomes was more manageable compared to that of the unbound drugs BH. In the anti-tumor experiments, the incorporation of MT effectively promoted the anti-tumor effect of BH and exerted inhibitory effects on three types of cancer cells. This meant that the combined use of BH and MT could improve the efficacy of drugs (BH-MT > BH; BH-MT-LP > BH-LP). Moreover, the encapsulation of these drugs in liposomes can also enhance anti-tumor activity (BH-MT-LP > BH-MT; BH-LP > BH). The mechanism of action might involve liposomes enhancing the stability of BH and providing a sustained release effect. The study of its mechanism of action revealed that BH-MT-LP displayed a more potent inhibitory effect on the proliferation of MDA-MB-231 cancer cells, leading to a greater degree of cell cycle inhibition and induction of apoptosis. We were also motivated by findings that drugs encapsulated in liposomes could be structurally modified to achieve organ targeting. The concept of the compatibility of traditional Chinese medicine was developed into the combined use of Chinese medicine components, and the mechanism of action of Chinese medicine was studied with modern technology. To sum up, BH-MT-LP exhibits enormous anti-tumor potential, establishing a basis for boosting the therapeutic efficacy of BH and broadening its scope of application.

## Figures and Tables

**Table 1:** Comparison of EE of different preparation methods.

Preparation Method	EE (%)	DL (%)
Thin-film dispersion method	86.91	6.29
Reverse evaporation method	94.17	6.99
Ether injection method	91.74	6.44
Ammonium sulfate gradient method	79.33	5.94

**Table 2:** BBD design and results table.

Group	A	B	C	EE (%)	DL (%)
1	10	1:1	3	90.12	6.42
2	10	1:1	3	89.97	6.43
3	5	1:2	3	81.49	3.41
4	10	3:2	5	78.58	6.05
5	10	1:1	3	92.47	6.77
6	10	1:2	1	84.66	6.09
7	5	3:2	3	91.9	3.61
8	15	1:1	5	76.05	7.93
9	10	3:2	1	91.35	6.51
10	5	1:1	1	92.93	3.95
11	5	1:1	5	90.66	3.83
12	15	3:2	3	74.65	7.86
13	15	1:1	1	80.11	8.31
14	10	1:1	3	87.4	6.57
15	10	1:1	3	87.95	6.57
16	15	1:2	3	83.61	8.98
17	10	1:2	5	88.05	6.63

**Table 3:** Variance analysis of EE regression model.

Source	Sum of Squares	df	Mean Square	F-Value	*p*-Value
Model	524.64	9	58.29	10.59	0.0026
A-A	226.42	1	226.42	41.15	0.0004
B-B	0.2211	1	0.2211	0.0402	0.8468
C-C	30.85	1	30.85	5.61	0.0498
AB	93.8	1	93.8	17.05	0.0044
AC	0.801	1	0.801	0.1456	0.7141
BC	65.29	1	65.29	11.87	0.0108
A^2^	57.52	1	57.52	10.45	0.0144
B^2^	37.23	1	37.23	6.77	0.0354
C^2^	3.79	1	3.79	0.6884	0.4341
Residual	38.52	7	5.5		
Lack of Fit	22.31	3	7.44	1.84	0.2809
Pure Error	16.21	4	4.05		
Cor Total	563.16	16			

**Table 4:** Variance analysis of DL regression model.

Soruce	Sum of Squares	df	Mean Square	F-Value	*p*-Value
Model	43.67	9	4.85	79.75	<0.0001
A-A	41.77	1	41.77	686.47	<0.0001
B-B	0.1458	1	0.1458	2.4	0.1656
C-C	0.0221	1	0.0221	0.3624	0.5662
AB	0.4356	1	0.4356	7.16	0.0317
AC	0.0169	1	0.0169	0.2777	0.6145
BC	0.25	1	0.25	4.11	0.0823
A^2^	0.8564	1	0.8564	14.08	0.0072
B^2^	0.0779	1	0.0779	1.28	0.2952
C^2^	0.0388	1	0.0388	0.6377	0.4508
Residual	0.4259	7	0.0608		
Lack of Fit	0.3455	3	0.1152	5.72	0.0626
Pure Error	0.0805	4	0.0201		
Cor Total	44.1	16			

**Table 5:** Comparison of the predicted and experimental values.

Samples	EE (%)	DL (%)
Verification 1	88.87	6.95
Verification 2	87.95	7.22
Verification 3	90.84	6.84
Average value	89.22	7.03
Predicted value	89.97	7.01
Deviation value	−0.0084	0.0028

**Table 6:** Chromatographic conditions.

Time	A	B
0–6 min	90%	10%
6–20 min	90–35%	10–65%
20–25 min	35–20%	65–80%
25–35 min	20%	80%
35–36 min	20–90%	80–10%
36–42 min	90%	10%

## Data Availability

The data presented in this study are available upon request from the corresponding author.
